# The Causal Relationship between Endothelin-1 and Hypertension: Focusing on Endothelial Dysfunction, Arterial Stiffness, Vascular Remodeling, and Blood Pressure Regulation

**DOI:** 10.3390/life11090986

**Published:** 2021-09-20

**Authors:** Krasimir Kostov

**Affiliations:** Department of Pathophysiology, Medical University-Pleven, 1 Kliment Ohridski Str., 5800 Pleven, Bulgaria; dr.krasi_kostov@abv.bg; Tel.: +359-889-257-459

**Keywords:** endothelin-1, hypertension, oxidative stress, low-grade inflammation, endothelial dysfunction, arterial stiffness, arterial remodeling, blood pressure regulation

## Abstract

Hypertension (HTN) is one of the most prevalent diseases worldwide and is among the most important risk factors for cardiovascular and cerebrovascular complications. It is currently thought to be the result of disturbances in a number of neural, renal, hormonal, and vascular mechanisms regulating blood pressure (BP), so crucial importance is given to the imbalance of a number of vasoactive factors produced by the endothelium. Decreased nitric oxide production and increased production of endothelin-1 (ET-1) in the vascular wall may promote oxidative stress and low-grade inflammation, with the development of endothelial dysfunction (ED) and increased vasoconstrictor activity. Increased ET-1 production can contribute to arterial aging and the development of atherosclerotic changes, which are associated with increased arterial stiffness and manifestation of isolated systolic HTN. In addition, ET-1 is involved in the complex regulation of BP through synergistic interactions with angiotensin II, regulates the production of catecholamines and sympathetic activity, affects renal hemodynamics and water–salt balance, and regulates baroreceptor activity and myocardial contractility. This review focuses on the relationship between ET-1 and HTN and in particular on the key role of ET-1 in the pathogenesis of ED, arterial structural changes, and impaired vascular regulation of BP. The information presented includes basic concepts on the role of ET-1 in the pathogenesis of HTN without going into detailed analyses, which allows it to be used by a wide range of specialists. Also, the main pathological processes and mechanisms are richly illustrated for better understanding.

## 1. Introduction

Hypertension (HTN) is one of the most prevalent socially significant diseases and is among the most important preventable risk factors for other diseases [1]. The heart, brain, kidneys, and peripheral arteries are often affected, which is a cause of early disability and reduced life expectancy in patients [2]. This necessitates that the prevention and treatment of HTN be among the top priorities of public health worldwide [3]. 

HTN is a heterogeneous disease with a complex pathogenesis. It is currently thought to be the result of disturbances in a number of neural, renal, hormonal, and vascular mechanisms regulating blood pressure (BP) [4], as crucial importance is given to the imbalance of a number of vasoactive substances, some of which are produced from the vascular endothelium [5]. The endothelium responds to humoral, neural, and especially hemodynamic stimuli, and regulates platelet function, inflammatory responses, growth and migration of vascular smooth muscle cells (VSMCs), and changes in the structure of the vascular extracellular matrix [6,7]. In addition to these functions, it modulates vascular tone by synthesizing and releasing a number of vasoactive factors that may have vasodilatory effects, such as nitric oxide (NO), prostacyclin (PGI_2_), and endothelium-derived hyperpolarizing factor, and vasoconstrictor effects, such as thromboxane A_2_ and endothelin-1 (ET-1) [8]. In HTN, the delicate balance between vasodilators and vasoconstrictors is disturbed, leading to endothelial dysfunction (ED) with excessive release of vasoconstrictor substances, such as ET-1 [9,10]. 

**Figure 1:**
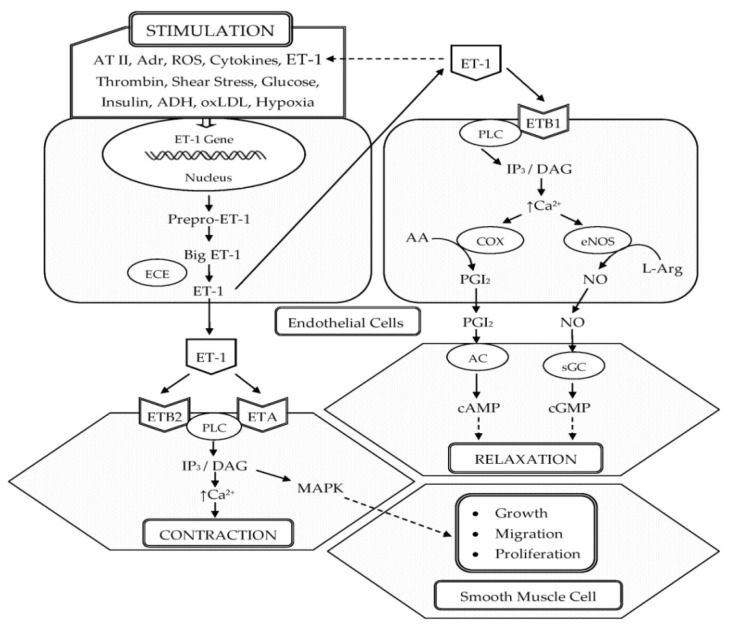
Schematic representation of the biosynthesis and vascular effects of ET-1 in endothelial and smooth muscle cells. ET-1 is generated by ECs in response to different stimuli. ET-1 mediates vasoconstriction by activating ETA and ETB2 receptors on VSMCs. Vasodilation by ET-1 is mediated through the activation of ETB1 on ECs, which increases the production of NO and PGI_2_. In addition, ET-1 is a potent mitogen that simulates the growth, proliferation, and migration of VSMCs. Abbreviations: ET-1, endothelin-1; AT II, angiotensin II; Adr, adrenaline; ROS, reactive oxygen species; ADH, antidiuretic hormone; oxLDL, oxidized low-density lipoproteins; ECE, endothelin-converting enzyme; ETA, endothelin receptor subtype A; ETB1, endothelin receptor subtype B1; ETB2 endothelin receptor subtype B2; PLC, phospholipase C; IP3, inositol trisphosphate; DAG, diacylglycerol; Ca^2+^, calcium ions; COX, cyclooxygenase; AA, arachidonic acid; PGI_2_, prostacyclin; eNOS, endothelial nitric oxide synthase; L-Arg, L-arginine; NO, nitric oxide; AC, adenylate cyclase; cAMP, cyclic adenosine monophosphate; sGC, soluble guanylate cyclase; cGMP, cyclic guanosine monophosphate; MAPK, mitogen-activated protein kinase; ↑, increased.

ET-1 was first isolated in 1988 by Yanagisawa and colleagues from the culture supernatant of porcine aortic endothelial cells (ECs). It is composed of 21 amino acids and two intrachain disulfide linkages in the molecule [11]. Shortly after the discovery of ET-1, two other structurally similar isopeptides, named ET-2 and ET-3, were isolated [12]. ET-1 is the predominant isopeptide involved in regulating the cardiovascular system, and vascular ECs are the most abundant source of ET-1. In addition to ECs, ET-1 is expressed in a wide variety of cells including VSMCs, cardiomyocytes, fibroblasts, macrophages, epithelial cells in the lungs and kidneys, neurons, and glial cells [13]. The endothelins (ETs) are produced from their corresponding approximately 200-residue prepropolypeptides that are encoded by three distinct genes. These peptides are converted into inactive 38- or 39-amino acid intermediates called Big ETs (Big ET-1, Big ET-2, and Big ET-3) by furin-like endopeptidase. The Big ETs are then activated via proteolytic cleavage by the ET-converting enzymes (ECEs), ECE-1 and ECE-2 [14] (Figure 1). 

In the vasculature, ET-1 acts on ETA and ETB (ETB1 and ETB2) receptors located on the VSMCs and ECs to induce vascular contraction or vasodilation [15]. Vasoconstrictive action of ET-1 is mainly mediated through ETA. ET-1–ETA interaction on VSMCs increases intracellular calcium, leading to the phosphorylation and activation of myosin light chain, which causes vasoconstriction [16,17]. In pathophysiological conditions, the expression of ETB2 on VSMCs is increased and ET-1–ETB2 interaction also promotes vasoconstriction [16]. ET-1 induces long-lasting vasoconstriction resulting from the slow dissociation rate from ET receptors [18]. In 40% of adults, a genetically prohypertensive phenotype is present or is the result of partially epigenetically mediated environmental effects. This is related to the predominance of vasoconstrictor actions of ET-1 mediated by ETA and ETB2 in VSMC, which is due to the hypertensive effect of increased endothelial expression of the ET-1 gene (*EDN1*) [19]. However, vasodilation by ET-1 is mediated through ETB1 on ECs, which increases the production of NO and PGI_2_ [17] (Figure 1).

ET-1 is continuously released from the endothelial constitutive pathway. Low levels of ET-1 promote vasodilatation, whereas higher and pathophysiological concentrations increase BP and total peripheral vascular resistance [13]. In healthy volunteers, low doses of ET-1 infused into the brachial artery cause vasodilatation, consistent with ETB-mediated release of vasodilators, but this was followed by sustained vasoconstriction of the forearm vascular bed at higher doses because the peptide accessed the smooth muscle ETA receptors [20]. Thus, ETB-mediated release of NO and other vasodilators is crucial in acting as a counterregulatory pathway to limit ETA-mediated vasoconstriction. In pathophysiological conditions where there is ED with a loss of vasodilators, the vasoconstrictor and other pathophysiological effects of ET-1, such as cell proliferation, will be potentiated [13]. In patients with essential HTN, the activity of exogenous ET-1 is increased, similar, or decreased compared to normotensive subjects, depending on which vascular district or scheme of administration is considered [21].

## 2. Data on the Participation of ET-1 in the Development of HTN

Due to its ability to maintain basal vascular tone [22,23] and homeostasis of sodium and water [24,25], it is suggested that ET-1 is involved in some forms of HTN [26], which is supported by various experimental and clinical observations.

### 2.1. ET-1 in Experimental HTN

The role of ET-1 in HTN was initially observed in models of experimental HTN, and more recently by using genetically modified mouse models in which a component of the endothelin system was either knockdown or overexpressed in certain specific organs or tissues [27]. It should be noted that when ET-1 is overexpressed in the endothelium of transgenic mice, BP is significantly higher than in wild-type mice [28]. Moreover, selective knockout of ET-1 in the collecting duct of the nephron was associated with a higher BP and the development of salt-sensitive HTN [29]. A similar BP phenotype was obtained with the deletion of ETB or both ETA/ETB receptors in collecting duct cells, suggesting the important role of the renal endothelin system in the development of salt-sensitive HTN [30,31]. In non-transgenic animals, elevated ET-1 production was found in salt-sensitive and some other models of experimental HTN, such as deoxycorticosterone (DOCA)-salt HTN, DOCA-salt-treated spontaneously hypertensive rats (SHR), and Dahl salt-sensitive rats, 1-kidney 1 clip (1K1C) Goldblatt hypertensive rats, SHR 2-kidney 1 clip (2K1C) Goldblatt hypertensive rats, angiotensin II-infused rats, and stroke-prone SHR [32]. In the models known to overexpress vascular ET-1, BP significantly decreases upon the administration of selective ETA or mixed ETA/ETB receptor antagonists [33].

### 2.2. ET-1 in Human HTN 

One of the first comparisons of ET-1 concentrations in people with HTN was made between pheochromocytoma patients and healthy controls. Higher levels of ET-1 were observed in patients with pheochromocytoma. In this report, the authors note that HTN in patients with pheochromocytoma is mainly catecholamine dependent, but may be secondarily ET-1 dependent [34]. These data are supported by previously reported cases in patients with hemangioendothelioma who have significantly elevated ET-1 levels along with HTN [35]. Elevated ET-1 levels and high BP in patients from these studies returned to normal after surgical removal of the tumors [34,35]. In addition, resistant HTN with elevated ET-1 levels has been observed more frequently in patients of African-American descent or those with obesity, in whom the risk of developing cardiovascular and renal diseases is increased [36]. Furthermore, in individuals with normal BP, high plasma ET-1 levels are associated with the development of HTN [37]. The role of ET-1 in the development of the hypertensive process is also supported by data in patients with essential HTN or resistant HTN, which show that when treated with a nonselective ET-receptor antagonist bosentan [38] or with the selective ETA receptor antagonist darusentan [39,40,41], BP is significantly reduced. These data are also consistent with the meta-analyses, which show that HTN patients have higher plasma concentrations of ET-1 than control subjects [42]. Other authors have reported that the levels of ET-1 are normal in patients with essential HTN, but point out that the local levels of ET-1 in the vascular wall are elevated [36,43,44]. The controversial and not always consistent results regarding ET-1 concentrations in patients with HTN are probably related to two main reasons. The first is that its elimination from the blood is too fast (plasma half-life 1–2 min) [45]. The second is that the secretion of ET-1 by ECs is polarized mainly to the underlying VSMCs, leading to a minimal increase in its circulating levels [46]. Other possible causes of these disparate results are the specificity of the antibodies used in the immunoassay, the degree of cardiovascular damage, dietary salt intake, obesity, diabetes, and race [24]. All of the above findings support the hypothesis that ET-1 may have an important pathogenetic role in the development of HTN (Table 1). 

## 3. Role of ET-1 in the Pathogenesis of HTN

Based on the accumulating data from experimental and clinical studies, it can be assumed that there is a link between the increased biological activity of ET-1 and the development of HTN [44,55,56,57,58]. Probably, ET-1 is causally related to high BP through synergistic interactions of the following mechanisms: (1) participation in the development of oxidative stress and low-grade inflammation in the vascular wall with the occurrence of ED; (2) participation in the pathogenesis of arterial stiffness; (3) participation in the processes of arterial remodeling; (4) participation in the mechanisms regulating BP. 

### 3.1. Participation of ET-1 in the Development of Oxidative Stress and Low-Grade Inflammation in the Vascular Wall with the Occurrence of ED

**Figure 2:**
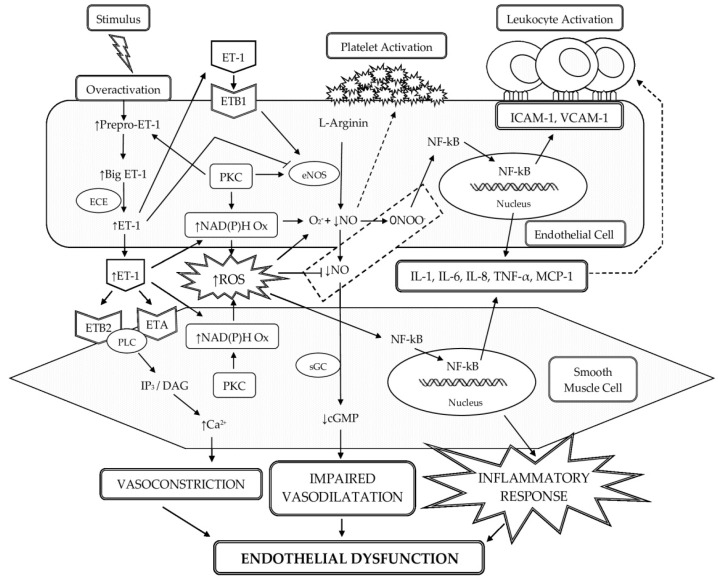
Schematic representation of the relationship of ET-1 with oxidative stress in the vascular wall, low-grade inflammation and ED. Increased production of ET-1 may decrease endothelial NO production by suppressing eNOS expression. In addition, ET-1 can mediate the formation of superoxide (O_2_^−^) by activating NAD(P)H oxidase, which may reduce the biological activity of NO in the vascular wall due to the formation of peroxynitrite (ONOO^−^). In ED, the increased production of ET-1 shifted the balance of effects toward increased vasoconstriction, oxidative stress, and inflammation. Abbreviations: ET-1, endothelin-1; ECE, endothelin-converting enzyme; ROS, reactive oxygen species; ETA, endothelin receptor subtype A; ETB1, endothelin receptor subtype B1; ETB2 endothelin receptor subtype B2; PLC, phospholipase C; IP3, inositol trisphosphate; DAG, diacylglycerol; eNOS, endothelial nitric oxide synthase; NO, nitric oxide; PKC, protein kinase C; NAD(P)H Ox, nicotinamide adenine dinucleotide phosphate oxidase; ROS, reactive oxygen species; O_2_^−^, superoxide anion; ONOO^−^, peroxynitrite; NF-κB, nuclear factor kappa B; IL-1, interleukin-1; IL-6, interleukin-6; IL-8, interleukin-8; TNF-α, tumor necrosis factor alpha; MCP-1, monocyte chemotactic protein-1; ICAM-1, intercellular adhesion molecule-1; VCAM-1, vascular cell adhesion molecule 1; sGC, soluble guanylate cyclase; cGMP, cyclic guanosine monophosphate; Ca^2+^, calcium ions; ↑, increased, enhanced activity; ↓, decreased.

ET-1 is linked to the pathogenesis of HTN by means of oxidative stress in the vascular wall [59,60,61,62] and low-grade vascular inflammation [63,64,65], which are the main drivers of ED (Figure 2). The relationship between oxidative stress in the vessel wall and the development of HTN is shown in many experimental models, including in human HTN [66,67,68,69]. Various studies support the role of ET-1 in the formation of reactive oxygen species (ROS) and its relationship with oxidative stress and ED in humans. ET-1 stimulates the production of ROS in human endothelial and vascular smooth muscle cell cultures [70,71], as well as in isolated vessels [72,73,74]. It is assumed that the main mechanism for the increased production of ROS in HTN is increased expression of vascular NAD(P)H oxidase [60,62,75,76,77]. Increased production of ROS in the vascular wall leads to the activation of nuclear factor kappa B. This, in turn, stimulates the synthesis of pro-inflammatory cytokines, chemokines, and adhesion molecules, which are associated with the development of vascular inflammatory response. Low-grade inflammation localized in the vascular tissue is an important factor in the pathophysiology of HTN [78,79,80]. Actually, oxidative stress and inflammation form a vicious cycle in the development of ED, which is implemented with active participation of ET-1 [81,82,83]. ET-1 can activate macrophages that lead to the release of pro-inflammatory and chemotactic mediators, such as tumor necrosis factor alpha, interleukin (IL)-1, IL-6, and IL-8 [84,85,86,87]. In turn, these pro-inflammatory cytokines can stimulate the production of ET-1 [88], and this could lead to increased BP [51,89,90]. It is assumed that under physiological conditions, the vasodilating action of ET-1 may predominate, whereas under pathophysiological conditions, ET-1 may behave as a vasoconstrictor and play a role in the pathophysiology of HTN [19]. 

### 3.2. Participation of ET-1 in the Pathogenesis of Arterial Stiffness

**Figure 3:**
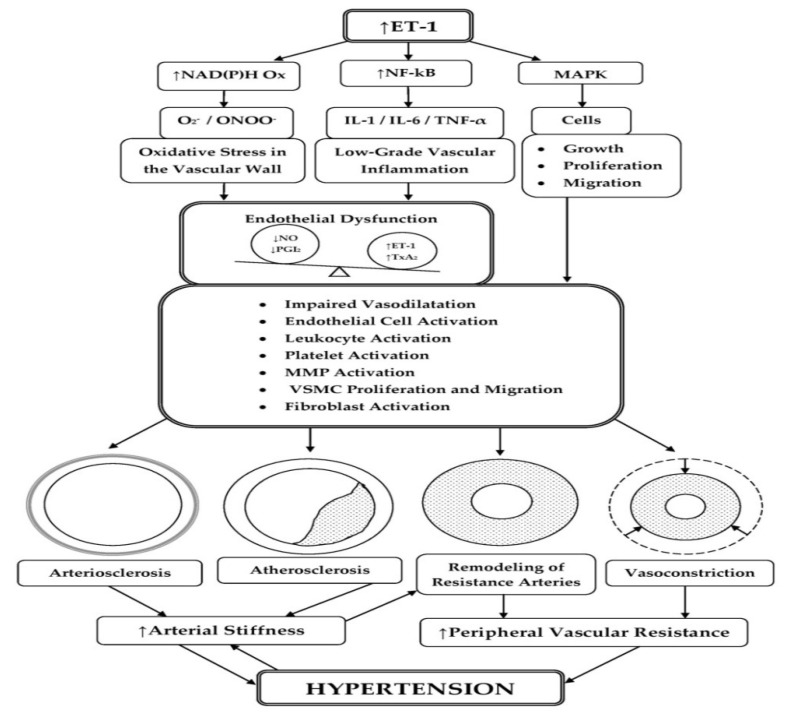
Schematic representation of the relationship of ET-1 with arterial stiffness and arterial remodeling as factors for the occurrence of HTN. Increased ET-1 activity may contribute to arterial stiffness in arteriosclerosis and atherosclerosis. These pathological processes significantly reduce the elastic properties of the central conduit arteries, which leads to the manifestation of isolated systolic HTN. Increased systolic and central pulse pressure may lead to eutrophic or hypertrophic remodeling of the small arteries. In particular, hypertrophic remodeling of resistance arteries is a signature of involvement of ET-1 in the hypertensive process. Abbreviations: ET-1, endothelin-1; NO, nitric oxide; PGI_2_, prostacyclin; NAD(P)H Ox, nicotinamide adenine dinucleotide phosphate oxidase; O_2_^−^, superoxide anion; ONOO^−^, peroxynitrite; NF-κB, nuclear factor kappa B; IL-1, interleukin-1; IL-6, interleukin-6; TNF-α, tumor necrosis factor alpha; MAPK, mitogen-activated protein kinase; TxA_2_, thromboxane A_2_; ↑, increased, enhanced activity; ↓, decreased.

A number of experimental and clinical studies have shown that ET-1 is responsible for maintaining arterial stiffness [27,91,92]. In ED, where the production of NO is reduced and that of ET-1 is increased, the balance is changed to increase arterial stiffness [57]. Arteriosclerosis [93,94,95] and its most common form, atherosclerosis [96,97,98], are the main pathological processes associated with increased hardness of the arteries (Figure 3). They significantly reduce the elastic properties of the arterial wall, which leads to an increase in the pulse wave velocity of the large conductive arteries [99,100] and to the manifestation of isolated systolic HTN [101,102].

#### 3.2.1. Role of ET-1 in Arteriosclerosis

Arteriosclerosis is a pathological process of arterial aging that results from interactive genetic and epigenetic events within different cell types of vascular tissue and its extracellular matrix [95,103,104,105]. Increased ET-1 activity can contribute to vascular dysfunction and arterial aging through multiple pathways, such as direct hemodynamic effects, vascular oxidative stress, inflammatory activity, mitogenic VSMC stimulation, and fibrotic processes [106]. It has been found that the vasoconstrictor activity of ET-1 increases in the elderly [107,108] and that the synthesis of ET-1 is greater in cultured aortic ECs obtained from older compared with younger donors [109,110]. In addition, ET-1 can stimulate collagen synthesis by fibroblasts and the development of vascular fibrosis by activating ETA and ETB receptors [111,112,113]. In addition, ET-1 mediates transforming growth factor beta activation, which can further induce fibrosis [114].

#### 3.2.2. Role of ET-1 in Atherosclerosis

Atherosclerosis is a specific type of arteriosclerosis that is characterized by the build-up of intimal plaques inside the arteries and narrowing of their lumen. Previous studies on experimental animal models and humans have shown a key role of ET-1 in the pathogenesis of atherosclerosis [115,116,117,118,119,120,121]. In one classic experiment in which mice overexpressing human pre-proET-1 in the endothelium (eET-1 mice) have been crossed with atherosclerosis-prone mice (apolipoprotein E −/− mice) and fed a high-fat diet, the lipid-containing plaques in crossed animals (eET-1/apolipoprotein E −/−) have been increased dramatically more than in E −/− mice, along with an increase in BP. These findings suggest that increased endothelial expression of ET-1 accelerates the progression of atherosclerosis and may be the link between atherosclerosis and HTN [120]. Increased expression of ET-1 was also observed in human arteries at various stages of atherosclerosis [122]. Plasma concentrations of ET-1 also showed a positive correlation with the stages of atherosclerosis [117]. In addition, the expression of Big ET-1 and ECE-1 was increased in atherosclerotic arteries [123], and the ETA and ETB receptors were highly expressed in smooth muscle cells and foam macrophages at the sites of atherosclerotic lesions [124]. ET-1 may also be involved in the inflammatory process and migration of VSMCs, as well as in the phenotypic transformation of VSMCs into proliferative synthetic cells that produce the extracellular matrix of the plaque [125].

### 3.3. Participation of ET-1 in the Processes of Arterial Remodeling

In HTN, the change in the structure of resistance arteries involves two processes: inward eutrophic remodeling and hypertrophic remodeling [126]. In eutrophic remodeling, the outer diameter and the lumen are decreased and the cross-sectional area of the media is unaltered. This type of remodeling predominates in resistant arteries of SHR and 2K1C Goldblatt hypertensive rats in which the renin–angiotensin system plays an important role. In humans, eutrophic remodeling is found in mild, essential HTN. In contrast, hypertrophic remodeling involves increased medial cross-sectional area and decreased lumen [127]. Hypertrophic remodeling of resistance arteries has been a characteristic finding in most rat models of severe HTN in which the endothelin system is activated [128,129], such as deoxycorticosterone (DOCA)-salt hypertensive rats [130], 1-kidney 1 clip (1K1C) Goldblatt hypertensive rats [131], and Dahl salt-sensitive HTN [132]. In humans, it can be found in secondary HTN, for example, in renovascular HTN or HTN associated with pheochromocytoma [133]. ET-1 plays an important role in abnormal vascular function and remodeling of resistance arteries [27] (Figure 3). ET-1 has a direct hypertrophic effect on the vasculature, in particular on the small arteries, and hypertrophic remodeling is a sign of involvement of ET-1 in the hypertensive process [133]. 

### 3.4. Participation of ET-1 in the Mechanisms of BP Regulation

**Figure 4:**
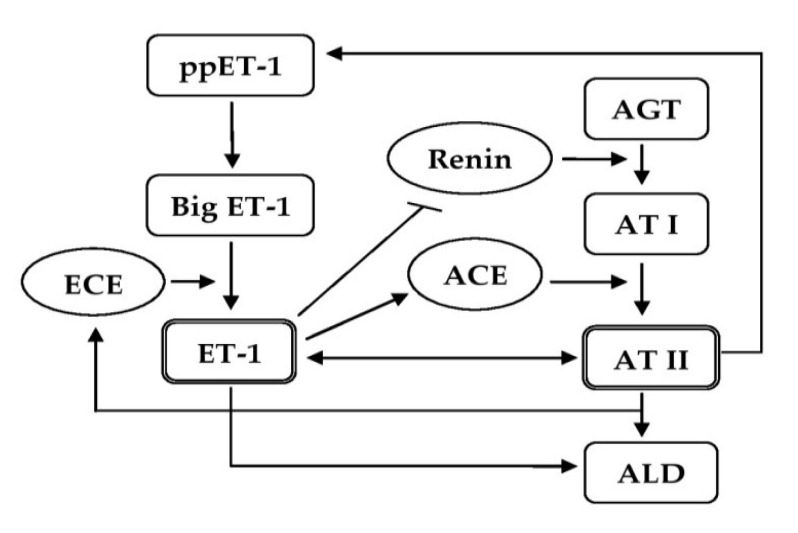
Schematic representation of synergistic interactions between ET-1 and AT II in the pathogenesis of HTN. ET-1 increases the formation of AT II by enhancing ACE activity. In turn, AT II increases the synthesis of ET-1 by enhancing ECE activity. ET-1 inhibits renin release, but can directly stimulate ALD production. Abbreviations: ET-1, endothelin-1; ppET-1, prepro-ET-1; ECE, endothelin-converting enzyme; AGT, angiotensinogen; AT I, angiotensin I; AT II, angiotensin II; ACE, angiotensin-converting enzyme; ALD, aldosterone.

**Figure 5:**
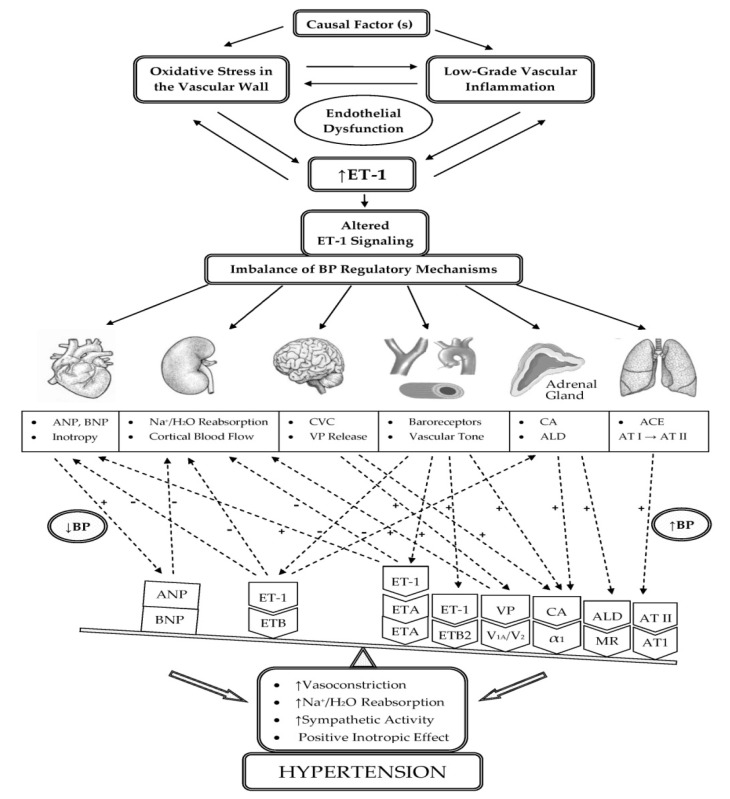
Schematic representation of integrated regulatory effects of ET-1 in HTN. Excessive activation of ET-1 leads to an increase in BP by enhancing the effects of ET-1/ETA signaling in the vasculature, adrenal gland, kidney, nervous system, and heart, which predominate over the BP-lowering effects of ET-1/ETB signaling. The cumulative effect is increased vasoconstriction, increased Na^+^ and H_2_O reabsorption, increased sympathetic activity, and a positive inotropic effect on the myocardium, which may lead to the development of HTN. Abbreviations: ET-1, endothelin-1; ETA, endothelin receptor subtype A; ETB, endothelin receptor subtype B; ETB2, endothelin receptor subtype B2; AT I, angiotensin I; AT II, angiotensin II; ACE, angiotensin-converting enzyme; AT1, angiotensin II receptor type 1; ANP, atrial natriuretic peptide; BNP, brain natriuretic peptide; VP, vasopressin; V_1A_, vasopressin 1A receptor; V_2_, vasopressin 2 receptor; CA, catecholamines; α_1_, alpha-1 adrenergic receptor; ALD, aldosterone; MR, mineralocorticoid receptor; CVC, cardiovascular center; H_2_O, water; Na^+^, sodium; +/−, activation/inhibition; ↑, increased.

BP regulation is an integrative process that involves complex interactions between the structures of the nervous system, cardiovascular system, hormones, and renal balance of fluids, which are in continuous feedback with specialized receptors related to monitoring the volume and hemodynamic parameters of blood circulation [134]. ET-1 can raise BP by disturbing some of these regulatory mechanisms (Figure 5), and in particular, maintaining intravascular fluid volume [24,25,135], peripheral vascular resistance [22,136,137], and cardiac contractility [138,139,140]. ET-1 is involved in maintaining intravascular volume by regulating the tubular reabsorption of water and electrolytes in the kidneys [24,25], affecting the production of aldosterone [141,142] and the secretion of vasopressin and natriuretic peptides [143,144]. ET-1 affects peripheral vascular resistance through its powerful vasoconstrictor effect, through the regulation of catecholamine secretion by the adrenal glands [145], as well as through its synergistic interactions with AT II [146,147] (Figure 4 and Figure 5). In a number of pathological processes, overstimulation of ET-1/ETA signaling may upset the balance in the regulation of these mechanisms, which may subsequently lead to the development of HTN [24]. 

In addition, ET-1 is involved in maintaining BP by regulating the cardiovascular center [148] and baroreceptor activity [149]. The paraventricular nucleus (PVN) is an important integrative center in the control of the cardiac sympathetic afferent reflex, which is a positive-feedback, sympathoexcitatory reflex. Abundant ET-1 expression is found in the PVN, especially in the parvocellular PVN cells [150]. The projections from the PVN to brain stem loci lead to increases in sympathetic efferent output and BP [24]. The lesion of the PVN prevents the intracerebroventricular administration of ET-1-induced increase in BP [151]. Projections from the PVN and area postrema also modulate the nucleus tractus solitarius, which, in turn, sends inputs to the PVN. Collectively, these regions function to regulate sympathetic and parasympathetic outflow to the heart and sympathetic output to the vasculature, kidney, and elsewhere. Interactions between these regions are complex, involving both excitatory and inhibitory signals. ET-1 and its receptors have been implicated in the enhanced sympathetic excitability observed in models of salt-sensitive HTN such as the DOCA-salt and ETB-deficient rats [24]. Endogenous ET-1 appears to have a sympathoexcitatory effect in both normotensive and hypertensive subjects through ETA receptors, contributing to basal sympathetic vasomotor tone. Moreover, HTN shows an increased susceptibility to the sympathoexcitatory effect of endogenous ET-1. The discovery of enhanced biological activity of ET-1 on autonomic cardiovascular regulation, beyond the known effects on vascular tone, further reinforces the fundamental role of the endothelin system in the pathophysiology of HTN. Thus, treatment options aimed at counteracting the effects of ET-1 could have a beneficial effect on the adrenergic overactivity observed in HTN [152]. 

## 4. ET-1 as a Potential Therapeutic Target in HTN

Considerable progress has been made during the last two decades in characterizing the pharmacology of the ET-1 signaling pathway with the development of key compounds, such as selective ETA and ETB receptor antagonists (ERAs), dual endothelin receptor/angiotensin receptor antagonists (DARAs), together with selective ETB receptor agonists and radiolabeled analogs to accurately describe the ET system and its role in human and animal models of HTN [13]. The development of orally active ERAs that had become available by the mid-1990s allowed to study whether and how endogenous ET-1 contributes to the pathophysiology of essential HTN. Treatment with bosentan, a nonselective ERA, or darusentan, an ETA-selective ERA, decreased arterial BP in patients with essential HTN. Antihypertensive efficacy in patients with essential hypertension has also been reported for the DARA sparsentan [153]. Aprocitentan is a novel, oral, dual ERA that has demonstrated a more favorable tolerability and safety profile in early clinical trials compared with other ERAs [154]. ERAs remain an important part of pulmonary arterial hypertension (PAH) treatment. Treatment with approved ERAs, such as bosentan, ambrisentan, and macitentan, slows down PAH progression and relieves symptoms. However, more studies are needed to assess the benefits and safety of ERA treatment in patients with arterial HTN [155]. ERAs could play a particular role in the treatment of high-risk patients, such as those with resistant and salt-sensitive hypertension, those with progressive chronic kidney disease, those who develop hypertension after transplantation, or those with hypertension as part of the metabolic syndrome or diabetes [26]. The general side effects of ERAs are related to the vasodilator properties, including flushing, nausea, headache, nasal congestion, and peripheral edema, as well as hypotension and palpitations. Peripheral edema can be observed with the use of ERAs. Reduced hemoglobin levels and anemia can also appear during ERA treatment, as well as a reversible, dose-dependent elevation in aminotransferases [155]. 

## 5. Conclusions

ET-1 is involved in the physiological regulation of BP, thus exerting its influence on various processes: (1) it regulates vascular homeostasis, (2) regulates renal–endocrine mechanisms maintaining sodium and water balance, (3) modulates systemic hemodynamics, (4) affects the stiffness of the arteries, and (5) activates the natriuretic peptide system of the heart in chronic volume overload. In HTN, these regulatory mechanisms are disbalanced due to enhanced ET-1/ETA signaling in the vasculature, adrenal gland, kidney, nervous system, and heart. Impaired regulation shifts the balance toward increased vasoconstriction, increased Na^+^ and H_2_O reabsorption, increased sympathetic activity, and increased strength of cardiac contraction, which may lead to an increase in BP. In the long term, to these effects is added the increased systolic arterial pressure as a result of the structural changes in central and resistance arteries potentiated by ET-1, which leads to a permanent increase in BP and the development of HTN.

## Figures and Tables

**Table 1:** Studies on the contribution of ET-1 to the hypertensive phenotype in humans.

Study	Results	Significance
Saito, 1990 [47]	Patients with essential HTN showed a significant elevation in the plasma ET-1 level compared with age-matched control subjects.	*p* < 0.01
Shichiri, 1990 [48]	Patients with essential HTN had significantly higher plasma ET-1 levels than normal subjects.	*p* < 0.025
Oishi, 1994 [34]	In patients with pheochromocytoma, the hypertensive group had higher ET-1 than the normotensive group. Elevated plasma ET-1 concentrations returned to normal levels after surgical resection of the tumor.	Higher, but NS
Parrinello, 1996 [49]	ET-1 levels were significantly higher in obese hypertensives and obese normotensives than in lean normotensives. In addition, ET-1 levels were significantly higher in obese hypertensives than in obese normotensives.	*p* < 0.05
Amoroso, 1996 [50]	Patients with HTN had significantly higher plasma ET-1 concentration than normal subjects.	*p* < 0.02
Schneider, 2000 [51]	Basal ET-1 was significantly higher in hypertensive than in normotensive subjects, both in venous and arterial samples. There was no significant difference between venous and arterial ET-1 concentrations.	*p* < 0.01
Parissis, 2001 [52]	Patients with HTN showed significantly higher levels of ET-1 compared with normotensive controls.	*p* < 0.01
Kostov, 2014 [53]	Serum levels of ET-1 are significantly higher in patients with mild and severe HTN compared to the control group.	*p* < 0.02
Gu, 2015 [54]	Plasma ET-1 levels were higher in hypertensives than in controls.	*p* < 0.001
Kostov, 2016 [55]	Serum ET-1 concentrations were significantly higher in hypertensive patients with type 2 diabetes than in prehypertensive patients with diabetes and healthy normotensive controls.	*p* < 0.05

Abbreviations: ET-1, endothelin-1; HTN, hypertension; NS, not significant.

## Data Availability

Not applicable.

## References

[1] Oparil S., Acelajado M.C., Bakris G.L., Berlowitz D.R., Cífková R., Dominiczak A.F., Grassi G., Jordan J., Poulter N.R., Rodgers A. (2018). Hypertension. Nat. Rev. Dis. Primers.

[2] Mensah G.A., Croft J.B., Giles W.H. (2003). The heart, kidney, and brain as target organs in hypertension. Curr. Probl. Cardiol..

[3] Mills K.T., Stefanescu A., He J. (2020). The global epidemiology of hypertension. Nat. Rev. Nephrol..

[4] Touyz R.M., Feldman R.D., Harrison D.G., Schiffrin E.L. (2020). A new look at the mosaic theory of hypertension. Can. J. Cardiol..

[5] Félétou M., Köhler R., Vanhoutte P.M. (2010). Endothelium-derived vasoactive factors and hypertension: Possible roles in pathogenesis and as treatment targets. Curr. Hypertens. Rep..

[6] Cahill P.A., Redmond E.M. (2016). Vascular endothelium–gatekeeper of vessel health. Atherosclerosis.

[7] Davis G.E., Senger D.R. (2005). Endothelial extracellular matrix: Biosynthesis, remodeling, and functions during vascular morphogenesis and neovessel stabilization. Circ. Res..

[8] Sandoo A., van Zanten J.J.V., Metsios G.S., Carroll D., Kitas G.D. (2010). The endothelium and its role in regulating vascular tone. Open Cardiovasc. Med. J..

[9] Konukoglu D., Uzun H. (2016). Endothelial dysfunction and hypertension. Hypertension.

[10] Masaki T., Sawamura T. (2006). Endothelin and endothelial dysfunction. Proc. Jpn. Acad. Ser. B Phys. Biol. Sci..

[11] Yanagisawa M., Kurihara H., Kimura S., Tomobe Y., Kobayashi M., Mitsui Y., Yazaki Y., Goto K., Masaki T. (1988). A Novel potent vasoconstrictor peptide produced by vascular endothelial cells. Nature.

[12] Inoue A., Yanagisawa M., Kimura S., Kasuya Y., Miyauchi T., Goto K., Masaki T. (1989). The human endothelin family: Three structurally and pharmacologically distinct isopeptides predicted by three separate genes. Proc. Natl. Acad. Sci. USA.

[13] Davenport A.P., Hyndman K.A., Dhaun N., Southan C., Kohan D.E., Pollock J.S., Pollock D.M., Webb D.J., Maguire J.J. (2016). Endothelin. Pharmacol. Rev..

[14] Emoto N. (2017). Endothelin receptor antagonist. Diagnosis and Treatment of Pulmonary Hypertension.

[15] Pollock D.M., Keith T.L., Highsmith R.F. (1995). Endothelin receptors and calcium signaling. FASEB J..

[16] Miyagawa K., Emoto N. (2014). Current state of endothelin receptor antagonism in hypertension and pulmonary hypertension. Ther. Adv. Cardiovasc. Dis..

[17] Hynynen M.M., Khalil R.A. (2006). The vascular endothelin system in hypertension–recent patents and discoveries. Recent Pat. Cardiovasc. Drug Discov..

[18] Maguire J.J., Davenport A.P. (2015). Endothelin receptors and their antagonists. Semin. Nephrol..

[19] Schiffrin E.L. (2018). Does endothelin-1 raise or lower blood pressure in humans?. Nephron.

[20] Kiowski W., Lüscher T., Linder L., Bühler F. (1991). Endothelin-1-induced vasoconstriction in humans. Reversal by calcium channel blockade but not by nitrovasodilators or endothelium-derived relaxing factor. Circulation.

[21] Taddei S., Virdis A., Ghiadoni L., Sudano I., Magagna A., Salvetti A. (2001). Role of endothelin in the control of peripheral vascular tone in human hypertension. Heart Fail. Rev..

[22] Webb D.J. (1995). Endogenous endothelin generation maintains vascular tone in humans. J. Hum. Hypertens..

[23] Haynes W.G., Webb D.J. (1994). Contribution of endogenous generation of endothelin-1 to basal vascular tone. Lancet.

[24] Kohan D.E., Rossi N.F., Inscho E.W., Pollock D.M. (2011). Regulation of blood pressure and salt homeostasis by endothelin. Physiol. Rev..

[25] Speed J.S., Fox B.M., Johnston J.G., Pollock D.M. (2015). Endothelin and renal ion and water transport. Semin. Nephrol..

[26] Dhaun N., Goddard J., Kohan D.E., Pollock D.M., Schiffrin E.L., Webb D.J. (2008). Role of endothelin-1 in clinical hypertension: 20 years on. Hypertension.

[27] Amiri F., Virdis A., Neves M.F., Iglarz M., Seidah N.G., Touyz R.M., Reudelhuber T.L., Schiffrin E.L. (2004). Endothelium-restricted overexpression of human endothelin-1 causes vascular remodeling and endothelial dysfunction. Circulation.

[28] Leung J.W.-C., Wong W.T., Koon H.W., Mo F.M., Tam S., Huang Y., Vanhoutte P.M., Chung S.S.M., Chung S.K. (2011). Transgenic mice over-expressing ET-1 in the endothelial cells develop systemic hypertension with altered vascular reactivity. PLoS ONE.

[29] Ahn D., Ge Y., Stricklett P.K., Gill P., Taylor D., Hughes A.K., Yanagisawa M., Miller L., Nelson R.D., Kohan D.E. (2004). Collecting duct–specific knockout of endothelin-1 causes hypertension and sodium retention. J. Clin. Investig..

[30] Ge Y., Bagnall A., Stricklett P.K., Strait K., Webb D.J., Kotelevtsev Y., Kohan D.E. (2006). Collecting duct-specific knockout of the endothelin B receptor causes hypertension and sodium retention. Am. J. Physiol. Renal Physiol..

[31] Ge Y., Bagnall A., Stricklett P.K., Webb D., Kotelevtsev Y., Kohan D.E. (2008). Combined knockout of collecting duct endothelin A and B receptors causes hypertension and sodium retention. Am. J. Physiol. Renal Physiol..

[32] Schiffrin E.L. (2000). Endothelin: Role in experimental hypertension. J. Cardiovasc. Pharmacol..

[33] Schiffrin E.L., Turgeon A., Deng L.Y. (1997). Effect of chronic ETA-selective endothelin receptor antagonism on blood pressure in experimental and genetic hypertension in rats. Br. J. Pharmacol..

[34] Oishi S., Sasaki M., Sato T. (1994). Elevated immunoreactive endothelin levels in patients with pheochromocytoma. Am. J. Hypertens..

[35] Yokokawa K., Tahara H., Kohno M., Murakawa K.I., Yasunari K., Nakagawa K., Hamada T., Otani S., Yanagisawa M., Takeda T. (1991). Hypertension associated with endothelin-secreting malignant hemangioendothelioma. Ann. Intern. Med..

[36] Barton M., Yanagisawa M. (2008). Endothelin: 20 years from discovery to therapy. Can. J. Physiol. Pharmacol..

[37] Kumagae S.I., Adachi H., Jacobs D.R., Hirai Y., Enomoto M., Fukami A., Otsuka M., Nanjo Y., Esaki E., Kumagai E. (2010). High level of plasma endothelin-1 predicts development of hypertension in normotensive subjects. Am. J. Hypertens..

[38] Krum H., Viskoper R.J., Lacourciere Y., Budde M., Charlon V. (1998). the effect of an endothelin-receptor antagonist, bosentan, on blood pressure in patients with essential hypertension. N. Engl. J. Med..

[39] Roumen N., Egon P., Siegfried E. (2002). Darusentan: An effective endothelina receptor antagonist for treatment of hypertension. Am. J. Hypertens..

[40] Black H.R., Bakris G.L., Weber M.A., Weiss R., Shahawy M.E., Marple R., Tannoury G., Linas S., Wiens B.L., Linseman J.V. (2007). Efficacy and safety of darusentan in patients with resistant hypertension: Results from a randomized, double-blind, placebo-controlled dose-ranging study. J. Clin. Hypertens..

[41] Bakris G.L., Lindholm L.H., Black H.R., Krum H., Linas S., Linseman J.V., Arterburn S., Sager P., Weber M. (2010). Divergent results using clinic and ambulatory blood pressures: Report of a darusentan-resistant hypertension trial. Hypertension.

[42] Xu M., Lu Y.P., Hasan A.A., Hocher B. (2017). Plasma ET-1 concentrations are elevated in patients with hypertension–meta-analysis of clinical studies. Kidney Blood Press. Res..

[43] Barton M., Shaw S., d’Uscio L.V., Moreau P., Lüscher T.F. (1997). Angiotensin II increases vascular and renal endothelin-1 and functional endothelin converting enzyme activity in vivo: Role of ETA receptors for endothelin regulation. Biochem. Biophys. Res. Commun..

[44] Pinto-Sietsma S.J., Paul M. (1998). A role for endothelin in the pathogenesis of hypertension: Fact or fiction?. Kidney Int..

[45] Al-Omari M.A., Khaleghi M., Mosley T.H., Morgenthaler N.G., Struck J., Bergmann A., Kullo I.J. (2011). Plasma C-terminal pro-endothelin-1 is associated with left ventricular mass index and aortic root diameter in African-American adults with hypertension. J. Hum. Hypertens..

[46] Wolpe A.G., Ruddiman C.A., Hall P.J., Isakson B.E. (2021). Polarized proteins in endothelium and their contribution to function. J.Vasc. Res..

[47] Saito Y., Nakao K., Mukoyama M., Imura H. (1990). Increased plasma endothelin level in patients with essential hypertension. N. Engl. J. Med..

[48] Shichiri M., Hirata Y., Ando K., Emori T., Ohta K., Kimoto S., Ogura M., Inoue A., Marumo F. (1990). Plasma endothelin levels in hypertension and chronic renal failure. Hypertension.

[49] Parrinello G., Scaglione R., Pinto A., Corrao S., Cecala M., Di Silvestre G., Amato P., Licata A., Licata G. (1996). Central obesity and hypertension: The role of plasma endothelin. Am. J. Hypertens..

[50] Amoroso A., Cossu M., Mariotti A., Guido F., Ferri G., De Rosa F., Sportelli G. (1996). Increased plasma levels of endothelin in patients with essential arterial hypertension. Eur. Rev. Med. Pharmacol. Sci..

[51] Schneider M.P., Hilgers K.F., Klingbeil A.U., John S., Veelken R., Schmieder R.E. (2000). Plasma endothelin is increased in early essential hypertension. Am. J. Hypertens..

[52] Parissis J.T., Venetsanou K.F., Mentzikof D.G., Kalantzi M.V., Georgopoulou M.V., Chrisopoulos N., Karas S.M. (2001). Plasma levels of soluble cellular adhesion molecules in patients with arterial hypertension. Correlations with plasma endothelin-1. Eur. J. Intern. Med..

[53] Kostov K., Dimitrova A., Grigoryan A., Tisheva S., Ruseva A., Atanasova M., Gospodinov C., Blazhev A. (2014). Changes in the serum levels of endothelin-1, matrix metalloproteinases-2,-9 and C-reactive protein in patients with mild and severe degree of arterial hypertension. C. R. Acad. Bulg. Sci..

[54] Gu X., Li H., Zhu X., Gu H., Chen J., Wang L., Harding P., Xu W. (2015). Inverse correlation between plasma adropin and ET-1 levels in essential hypertension: A cross-sectional study. Medicine.

[55] Kostov K., Blazhev A., Atanasova M., Dimitrova A. (2016). Serum concentrations of endothelin-1 and matrix metalloproteinases-2, -9 in pre-hypertensive and hypertensive patients with type 2 diabetes. Int. J. Mol. Sci..

[56] del Villar C.P., Alonso C.J.G., Feldstein C.A., Juncos L.A., Romero J.C. (2005). Role of endothelin in the pathogenesis of hypertension. Mayo Clinic Proceedings.

[57] Iglarz M., Schiffrin E.L. (2003). Role of endothelin-1 in hypertension. Curr. Hypertens. Rep..

[58] Rahman M.A., Jesmin S., Islam M.M., Sohael F., Hasan A.S.H., Zaedi S., Sultana S.N., Yamaguchi N., Kawano S., Okazaki O. (2015). Circulatory level of endothelin-1 and hypertension in rural women in Bangladesh: A potential association evidenced from a community based cross-sectional study. J. Hypertens..

[59] Skalska A.B., Pietrzycka A., Stępniewski M. (2009). Correlation of endothelin-1 plasma levels with plasma antioxidant capacity in elderly patients treated for hypertension. Clin. Biochem..

[60] Romero M., Jiménez R., Sánchez M., López-Sepúlveda R., Zarzuelo A., Tamargo J., Pérez-Vizcaíno F., Duarte J. (2010). Vascular superoxide production by endothelin-1 requires Src non-receptor protein tyrosine kinase and MAPK activation. Atherosclerosis.

[61] Piechota A., Polańczyk A., Gorąca A. (2010). Role of endothelin-1 receptor blockers on hemodynamic parameters and oxidative stress. Pharmacol. Rep..

[62] Touyz R.M., Rios F.J., Alves-Lopes R., Neves K.B., Camargo L.L., Montezano A.C. (2020). Oxidative stress: A unifying paradigm in hypertension. Can. J. Cardiol..

[63] Böhm F., Pernow J. (2007). The importance of endothelin-1 for vascular dysfunction in cardiovascular disease. Cardiovasc. Res..

[64] Pernow J., Shemyakin A., Böhm F. (2012). New perspectives on endothelin-1 in atherosclerosis and diabetes mellitus. Life Sci..

[65] Tomiyama H., Shiina K., Matsumoto-Nakano C., Ninomiya T., Komatsu S., Kimura K., Chikamori T., Yamashina A. (2017). The contribution of inflammation to the development of hypertension mediated by increased arterial stiffness. J. Am. Heart Assoc..

[66] Vaziri N.D. (2004). Roles of oxidative stress and antioxidant therapy in chronic kidney disease and hypertension. Curr. Opin. Nephrol. Hypertens..

[67] Peterson J.R., Sharma R.V., Davisson R.L. (2006). Reactive oxygen species in the neuropathogenesis of hypertension. Curr. Hypertens. Rep..

[68] Harrison D.G., Gongora M.C. (2009). Oxidative stress and hypertension. Med. Clin. N. Am..

[69] Briones A.M., Touyz R.M. (2010). Oxidative stress and hypertension: Current concepts. Curr. Hypertens. Rep..

[70] Dong F., Zhang X., Wold L.E., Ren Q., Zhang Z., Ren J. (2005). Endothelin-1 enhances oxidative stress, cell proliferation and reduces apoptosis in human umbilical vein endothelial cells: Role of ETB receptor, NADPH oxidase and caveolin-1. Br. J. Pharmacol..

[71] Duerrschmidt N., Wippich N., Goettsch W., Broemme H.J., Morawietz H. (2000). Endothelin-1 induces NAD(P)H oxidase in human endothelial cells. Biochem. Biophys. Res. Commun..

[72] Galle J., Lehmann-Bodem C., Hübner U., Heinloth A., Wanner C. (2000). CyA and OxLDL cause endothelial dysfunction in isolated arteries through endothelin-mediated stimulation of O_2_^−^ formation. Nephrol. Dial. Transplant..

[73] Loomis E.D., Sullivan J.C., Osmond D.A., Pollock D.M., Pollock J.S. (2005). Endothelin mediates superoxide production and vasoconstriction through activation of NADPH oxidase and uncoupled nitric-oxide synthase in the rat aorta. J. Pharmacol. Exp. Ther..

[74] López-Sepúlveda R., Gómez-Guzmán M., Zarzuelo M.J., Romero M., Sánchez M., Quintela A.M., Galindo P., O’Valle F., Tamargo J., Pérez-Vizcaíno F. (2011). Red wine polyphenols prevent endothelial dysfunction induced by endothelin-1 in rat aorta: Role of NADPH oxidase. Clin. Sci..

[75] Mohazzab K., Kaminski P.M., Wolin M.S. (1994). NADH oxidoreductase is a major source of superoxide anion in bovine coronary artery endothelium. Am. J. Physiol. Heart Circ. Physiol..

[76] Kamata K., Kanie N., Matsumoto T., Kobayashi T. (2004). Endothelin-1-induced impairment of endothelium-dependent relaxation in aortas isolated from controls and diabetic rats. J. Cardiovasc. Pharmacol..

[77] Kanie N., Kamata K. (2002). Effects of chronic administration of the novel endothelin antagonist J-104132 on endothelial dysfunction in streptozotocin-induced diabetic rat. Br. J. Pharmacol..

[78] Camici G.G., Sudano I., Noll G., Tanner F.C., Lüscher T.F. (2009). Molecular pathways of aging and hypertension. Curr. Opin. Nephrol. Hypertens..

[79] Savoia C., Sada L., Zezza L., Pucci L., Lauri F.M., Befani A., Alonzo A., Volpe M. (2011). Vascular inflammation and endothelial dysfunction in experimental hypertension. Int. J. Hypertens..

[80] Barrows I.R., Ramezani A., Raj D.S. (2019). Inflammation, immunity, and oxidative stress in—partners in crime?. Adv. Chronic Kidney Dis..

[81] Guzik T.J., Touyz R.M. (2017). Oxidative stress, inflammation, and vascular aging in hypertension. Hypertension.

[82] Kähler J., Mendel S., Weckmüller J., Orzechowski H.D., Mittmann C., Köster R., Paul M., Meinertz T., Münzel T. (2000). Oxidative stress increases synthesis of big endothelin-1 by activation of the endothelin-1 promoter. J. Mol. Cell. Cardiol..

[83] Anggrahini D.W., Emoto N., Nakayama K., Widyantoro B., Adiarto S., Iwasa N., Nonaka H., Rikitake Y., Kisanuki Y.Y., Yanagisawa M. (2009). Vascular endothelial cell-derived endothelin-1 mediates vascular inflammation and neointima formation following blood flow cessation. Cardiovasc. Res..

[84] Ruetten H., Thiemermann C. (1997). Endothelin-1 stimulates the biosynthesis of tumour necrosis factor in macrophages: ET-receptors, signal transduction and inhibition by dexamethasone. J. Physiol. Pharmacol..

[85] Hofman F.M., Chen P., Jeyaseelan R., Incardona F., Fisher M., Zidovetzki R. (1998). Endothelin-1 induces production of the neutrophil chemotactic factor interleukin-8 by human brain-derived endothelial cells. Blood.

[86] Browatzki M., Schmidt J., Kübler W., Kranzhöfer R. (2000). Endothelin-1 induces interleukin-6 release via acctivation of the transcription factor NF-κB in human vascular smooth muscle cells. Basic Res. Cardiol..

[87] Yang L.L., Gros R., Kabir M.G., Sadi A., Gotlieb A.I., Husain M., Stewart D.J. (2004). Conditional cardiac overexpression of endothelin-1 induces inflammation and dilated cardiomyopathy in mice. Circulation.

[88] Virdis A., Schiffrin E.L. (2003). Vascular Inflammation: A role in vascular disease in hypertension?. Curr. Opin. Nephrol. Hypertens..

[89] Vierhapper H., Wagner O., Nowotny P., Waldhäusl W. (1990). Effect of endothelin-1 in man. Circulation.

[90] Letizia C., Celi M., Cerci S., Scuro L., Delfini E., Subioli S., Caliumi C., D’Erasmo E. (2001). High circulating levels of adrenomedullin and endothelin-1 in obesity associated with arterial hypertension. Ital. Heart J..

[91] McEniery C.M., Qasem A., Schmitt M., Avolio A.P., Cockcroft J.R., Wilkinson I.B. (2003). Endothelin-1 regulates arterial pulse wave velocity in vivo. J. Am. Coll. Cardiol..

[92] Vuurmans T.J., Boer P., Koomans H.A. (2003). Effects of endothelin-1 and endothelin-1 receptor blockade on cardiac output, aortic pressure, and pulse wave velocity in humans. Hypertension.

[93] Lee H.Y., Oh B.H. (2010). Aging and arterial stiffness. Circ. J..

[94] Sun Z. (2015). Aging, arterial stiffness, and hypertension. Hypertension.

[95] O’Rourke M.F. (2007). Arterial aging: Pathophysiological principles. Vasc. Med..

[96] Palombo C., Kozakova M. (2016). Arterial stiffness, atherosclerosis and cardiovascular risk: Pathophysiologic mechanisms and emerging clinical indications. Vascul. Pharmacol..

[97] Kim H.L., Kim S.H. (2019). Pulse wave velocity in atherosclerosis. Front. Cardiovasc. Med..

[98] Van Popele N.M., Grobbee D.E., Bots M.L., Asmar R., Topouchian J., Reneman R.S., Hoeks A.P., van der Kuip D.A., Hofman A., Witteman J.C. (2001). Association between arterial stiffness and atherosclerosis: The Rotterdam Study. Stroke.

[99] Rogers W.J., Hu Y.L., Coast D., Vido D.A., Kramer C.M., Pyeritz R.E., Reichek N. (2001). Age-associated changes in regional aortic pulse wave velocity. J. Am. Coll. Cardiol..

[100] Sutton-Tyrrell K., Najjar S.S., Boudreau R.M., Venkitachalam L., Kupelian V., Simonsick E.M., Havlik R., Lakatta E.G., Spurgeon H., Kritchevsky S. (2005). Elevated aortic pulse wave velocity, a marker of arterial stiffness, predicts cardiovascular events in well-functioning older adults. Circulation.

[101] Laurent S., Boutouyrie P. (2020). Arterial stiffness and hypertension in the elderly. Front. Cardiovasc. Med..

[102] Wallace S.M.L., Yasmin, McEniery C.M., Mäki-Petäjä K.M., Booth A.D., Cockcroft J.R., Wilkinson I.B. (2007). Isolated systolic hypertension is characterized by increased aortic stiffness and endothelial dysfunction. Hypertension.

[103] Sawabe M. (2010). Vascular aging: From molecular mechanism to clinical significance. Geriatr. Gerontol. Int..

[104] Laurent S., Boutouyrie P., Lacolley P. (2005). Structural and genetic bases of arterial stiffness. Hypertension.

[105] Harvey A., Montezano A.C., Lopes R.A., Rios F., Touyz R.M. (2016). Vascular fibrosis in aging and hypertension: Molecular mechanisms and clinical implications. Can. J. Cardiol..

[106] Trindade M., Oigman W., Fritsch Neves M. (2017). Potential role of endothelin in early vascular aging. Curr. Hypertens. Rev..

[107] Guilder G.P.V., Westby C.M., Greiner J.J., Stauffer B.L., DeSouza C.A. (2007). Endothelin-1 vasoconstrictor tone increases with age in healthy men but can be reduced by regular aerobic exercise. Hypertension.

[108] Thijssen D.H.J., Rongen G.A., Van Dijk A., Smits P., Hopman M.T.E. (2007). Enhanced endothelin-1-mediated leg vascular tone in healthy older subjects. J. Appl. Physiol..

[109] Tokunaga O., Fan J., Watanabe T., Kobayashi M., Kumazaki T., Mitsui Y. (1992). Endothelin. Immunohistologic localization in aorta and biosynthesis by cultured human aortic endothelial cells. Lab. Investig..

[110] Seals D.R., Jablonski K.L., Donato A.J. (2011). Aging and vascular endothelial function in humans. Clin. Sci..

[111] Horstmeyer A., Licht C., Scherr G., Eckes B., Krieg T. (2005). Signalling and regulation of collagen I synthesis by ET-1 and TGF-β1. FEBS J..

[112] Hafizi S., Wharton J., Chester A.H., Yacoub M.H. (2004). Profibrotic effects of endothelin-1 via the ETA receptor in cultured human cardiac fibroblasts. Cell. Physiol. Biochem..

[113] Clozel M., Salloukh H. (2005). Role of endothelin in fibrosis and anti-fibrotic potential of bosentan. Ann. Med..

[114] Wermuth P.J., Li Z., Mendoza F.A., Jimenez S.A. (2016). Stimulation of transforming growth factor-β1-induced endothelial-to-mesenchymal transition and tissue fibrosis by endothelin-1 (ET-1): A novel profibrotic effect of ET-1. PLoS ONE.

[115] Barton M., Haudenschild C.C., d’Uscio L.V., Shaw S., Münter K., Lüscher T.F. (1998). Endothelin ETA receptor blockade restores NO-mediated endothelial function and inhibits atherosclerosis in apolipoprotein E-deficient mice. Proc. Natl. Acad. Sci. USA.

[116] Lerman A., Webster M.W., Chesebro J.H., Edwards W.D., Wei C.M., Fuster V., Burnett J.C. (1993). Circulating and tissue endothelin immunoreactivity in hypercholesterolemic pigs. Circulation.

[117] Lerman A., Edwards B.S., Hallett J.W., Heublein D.M., Sandberg S.M., Burnett J.C. (1991). Circulating and tissue endothelin immunoreactivity in advanced atherosclerosis. N. Engl. J. Med..

[118] Zeiher A.M., Ihling C., Pistorius K., Schächinger V., Schaefer H.E. (1994). Increased tissue endothelin immunoreactivity in atherosclerotic lesions associated with acute coronary syndromes. Lancet.

[119] Böhm F., Johansson B.L., Hedin U., Alving K., Pernow J. (2002). Enhanced vasoconstrictor effect of big endothelin-1 in patients with atherosclerosis: Relation to conversion to endothelin-1. Atherosclerosis.

[120] Li M.W., Mian M.O.R., Barhoumi T., Rehman A., Mann K., Paradis P., Schiffrin E.L. (2013). Endothelin-1 overexpression exacerbates atherosclerosis and induces aortic aneurysms in apolipoprotein E knockout mice. Arterioscler. Thromb. Vasc. Biol..

[121] Lerman A., Holmes D.R., Bell M.R., Garratt K.N., Nishimura R.A., Burnett J.C. (1995). Endothelin in coronary endothelial dysfunction and early atherosclerosis in humans. Circulation.

[122] Ihling C., Szombathy T., Bohrmann B., Brockhaus M., Schaefer H.E., Loeffler B.M. (2001). Coexpression of endothelin-converting enzyme-1 and endothelin-1 in different stages of human atherosclerosis. Circulation.

[123] Maguire J.J., Davenport A.P. (1998). Increased response to big endothelin-1 in atherosclerotic human coronary artery: Functional evidence for up-regulation of endothelin-converting enzyme activity in disease. Br. J. Pharmacol..

[124] Sica D.A. (2008). Endothelin receptor antagonism: What does the future hold?. Hypertension.

[125] Chen Y., Su X., Qin Q., Yu Y., Jia M., Zhang H., Li H., Pei L. (2020). New insights into phenotypic switching of VSMCs induced by hyperhomocysteinemia: Role of endothelin-1 signaling. Biomed. Pharmacother..

[126] Lyle A.N., Taylor W.R. (2019). The pathophysiological basis of vascular disease. Lab. Investig..

[127] Intengan H.D., Schiffrin E.L. (2000). Structure and mechanical properties of resistance arteries in hypertension. Hypertension.

[128] Schiffrin E.L. (2001). Role of endothelin-1 in hypertension and vascular disease. Am. J. Hypertens..

[129] Schiffrin E.L. (1999). Role of endothelin-1 in hypertension. Hypertension.

[130] Li J.S., Larivière R., Schiffrin E.L. (1994). Effect of a nonselective endothelin antagonist on vascular remodeling in deoxycorticosterone acetate-salt hypertensive rats. Evidence for a role of endothelin in vascular hypertrophy. Hypertension.

[131] Abdel-Sayed S., Nussberger J., Aubert J.F., Gohlke P., Brunner H.R., Brakch N. (2003). Measurement of plasma endothelin-1 in experimental hypertension and in healthy subjects. Am. J. Hypertens..

[132] Ikeda T., Ohta H., Okada M., Kawai N., Nakao R., Siegl P.K.S., Kobayashi T., Maeda S., Miyauchi T., Nishikibe M. (1999). Pathophysiological roles of endothelin-1 in Dahl salt-sensitive hypertension. Hypertension.

[133] Schiffrin E.L. (2012). Vascular remodeling in hypertension. Hypertension.

[134] Nishida Y., Tandai-Hiruma M., Kemuriyama T., Hagisawa K. (2012). Long-term blood pressure control: Is there a set-point in the brain?. J. Physiol. Sci..

[135] Speed J.S., Heimlich J.B., Hyndman K.A., Fox B.M., Patel V., Yanagisawa M., Pollock J.S., Titze J.M., Pollock D.M. (2015). Endothelin Endothelin-1 as a master regulator of whole-body Na+ homeostasis. FASEB J..

[136] Nohria A., Garrett L., Johnson W., Kinlay S., Ganz P., Creager M.A. (2003). Endothelin-1 and vascular tone in subjects with atherogenic risk factors. Hypertension.

[137] Haynes W.G., Ferro C.E., Webb D.J. (1995). Physiologic role of endothelin in maintenance of vascular tone in humans. J. Cardiovasc. Pharmacol..

[138] Drawnel F.M., Archer C.R., Roderick H.L. (2013). The role of the paracrine/autocrine mediator endothelin-1 in regulation of cardiac contractility and growth. Br. J. Pharmacol..

[139] Zolk O., Münzel F., Eschenhagen T. (2004). Effects of chronic endothelin-1 stimulation on cardiac myocyte contractile function. Am. J. Physiol. Heart Circ. Physiol..

[140] Piuhola J., Mäkinen M., Szokodi I., Ruskoaho H. (2003). Dual role of endothelin-1 via ETA and ETB receptors in regulation of cardiac contractile function in mice. Am. J. Physiol. Heart Circ. Physiol..

[141] Pecci A., Gomez-Sanchez C.E., de Bedners M.E., Lantos C.P., Cozza E.N. (1993). In vivo stimulation of aldosterone biosynthesis by endothelin: Loci of action and effects of doses and infusion rate. J. Steroid Biochem. Mol. Biol..

[142] Andreis P.G., Neri G., Tortorella C., Aragona F., Rossi G.P., Nussdorfer G.G. (2002). Mechanisms transducing the aldosterone secretagogue signal of endothelins in the human adrenal cortex. Peptides.

[143] Yamamoto T., Kimura T., Ota K., Shoji M., Inoue M., Sato K., Ohta M., Yoshinaga K. (1991). Central effects of endothelin-1 on vasopressin and atrial natriuretic peptide release and cardiovascular and renal function in conscious rats. J. Cardiovasc. Pharmacol..

[144] Rossi N.F. (2004). Regulation of vasopressin secretion by ETA and ETB receptors in compartmentalized rat hypothalamo-neurohypophysial explants. Am. J. Physiol. Endocrinol. Metab..

[145] Ohara-Imaizumi M., Kumakura K. (1991). Dynamics of the secretory response evoked by endothelin-1 in adrenal chromaffin cells. J. Cardiovasc. Pharmacol..

[146] Yoshida K., Yasujima M., Kohzuki M., Tsunoda K., Kudo K., Kanazawa M., Yabe T., Abe K., Yoshinaga K. (1991). Chronic synergistic effect of endothelin-1 and angiotensin II on blood pressure in conscious rats. J. Cardiovasc. Pharmacol..

[147] Imai T., Hirata Y., Emori T., Yanagisawa M., Masaki T., Marumo F. (1992). Induction of endothelin-1 gene by angiotensin and vasopressin in endothelial cells. Hypertension.

[148] Ferguson A.V., Smith P. (1990). Cardiovascular responses induced by endothelin microinjection into area postrema. Regul. Pept..

[149] Li D.P., Fan Z.Z., He R.R. (1998). Modulatory effects of endothelin on carotid baroreflex in anesthetized rats. Acta Physiol. Sin..

[150] Chen A.D., Xiong X.Q., Gan X.B., Zhang F., Zhou Y.B., Gao X.Y., Han Y. (2012). Endothelin-1 in paraventricular nucleus modulates cardiac sympathetic afferent reflex and sympathetic activity in rats. PLoS ONE.

[151] Rossi N.F., Chen H. (2001). PVN lesions prevent the endothelin 1-induced increase in arterial pressure and vasopressin. Am. J. Physiol. Endocrinol. Metab..

[152] Bruno R.M., Sudano I., Ghiadoni L., Masi L., Taddei S. (2011). Interactions between sympathetic nervous system and endogenous endothelin in patients with essential hypertension. Hypertension.

[153] Barton M., Yanagisawa M. (2019). Endothelin: 30 years from discovery to therapy. Hypertension.

[154] McCoy E.K., Lisenby K.M. (2021). Aprocitentan (a dual endothelin-receptor antagonist) for treatment-resistant hypertension. J. Cardiovasc. Pharmacol..

[155] Enevoldsen F.C., Sahana J., Wehland M., Grimm D., Infanger M., Krüger M. (2020). Endothelin receptor antagonists: Status quo and future perspectives for targeted therapy. J. Clin. Med..

